# Acquired thrombotic thrombocytopenic purpura following Pfizer COVID‐19 vaccination

**DOI:** 10.1002/jha2.342

**Published:** 2021-11-16

**Authors:** Mawaddah Alislambouli, Andy Veras Victoria, Jyoti Matta, Faye Yin

**Affiliations:** ^1^ Department of Medicine Jersey City Medical Center Jersey City New Jersey USA

**Keywords:** acquired thrombotic thrombocytopenic purpura, ADAMTS‐13, mRNA COVID‐19 vaccine

## Abstract

Acquired thrombotic thrombocytopenic purpura (aTTP) is a rare disease and has occasionally been described after vaccination, especially against viral agents. We present a case of a patient who presents with the classic pentad of TTP a few days after receiving the first dose of the mRNA Pfizer COVID‐19 vaccine. To our knowledge, this is the second report of a de novo TTP following mRNA Pfizer COVID‐19 vaccination.

## 1 INTRODUCTION

Acquired thrombotic thrombocytopenic purpura (aTTP) is a rare disease with an incidence of 3–10 cases per million adults per year. The disease is defined by a severe reduction in the activity of von Willebrand factor‐cleaving protease ADAMTS 13, which is mainly caused by the presence of inhibitory antibodies. It is characterized by the microvascular aggregation of platelets that cause thrombocytopenia, microangiopathic hemolytic anaemia, and sometimes organ damage, which were historically defined as a pentad, typically presenting in <10% of patients with aTTP [1]. ATTP has occasionally been described after vaccination, especially against viral agents. We present a case of a patient who presents with the classic pentad of TTP a few days after receiving the first dose of the mRNA Pfizer COVID‐19 vaccine.

## 2 CASE PRESENTATION

A 61‐year‐old Korean‐American man with no past medical history presented with confusion, fever, headache, emesis, dark urine, and leg ecchymosis that developed 5 days following his first dose of Pfizer COVID 19 vaccine. Physical examination was notable for altered mentation, pallor, scleral icterus and bilateral lower extremity petechiae and ecchymosis. He developed a witnessed generalised tonic seizure and was intubated for airway protection at the emergency department.

**FIGURE 1:**
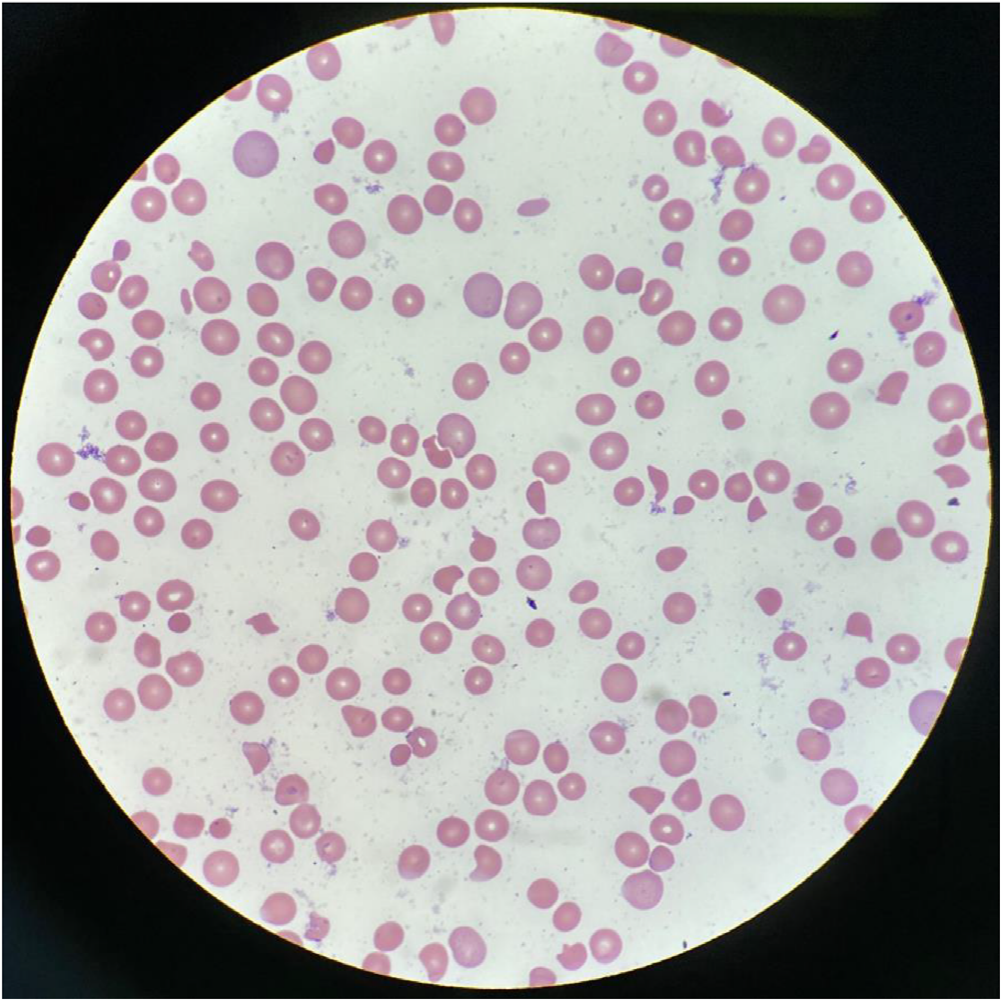
Patient peripheral smear

Laboratory revealed hemoglobin 65 g/L, platelets 6 × 10^9^/L, leukocytes 7 × 10^9^/L, creatinine 1.57 mg/dl, urea 47 mg/dl, total bilirubin 3.9 mg/dl, indirect bilirubin 3 mg/dl, LDH 1,757 UI/L, fibrinogen 490 mg/dl, haptoglobin < 8 mg/dl, D‐dimer 2.19 mcg/ml and reticulocytes 8%. Serum HIV and hepatitis panel were negative, COVID‐19 antigen, SARS‐CoV2 rapid and influenza antigen negative. Peripheral blood smear reveals schistocytes, more than 8 per high power field (Figure 1).

Given his fever, altered mentation, neurological deficits, evidence of hemolysis, thrombocytopenia, kidney injury and a PLASMIC score of six, he received emergent therapeutic plasmapheresis (FFP 1.5 × total plasma volume) in combination with methylprednisolone (1000 mg daily for three consecutive days). TTP was confirmed with an ADAMTS13 activity level of less than three. Initial CT head, CT angiogram of head and neck obtained before seizure episode were unremarkable. Repeat brain imaging following the seizure episode revealed a new small right parietal subdural hematoma 5 mm depth which did not increase in size. He remained seizure‐free and was extubated on the third day of admission.

**FIGURE 2:**
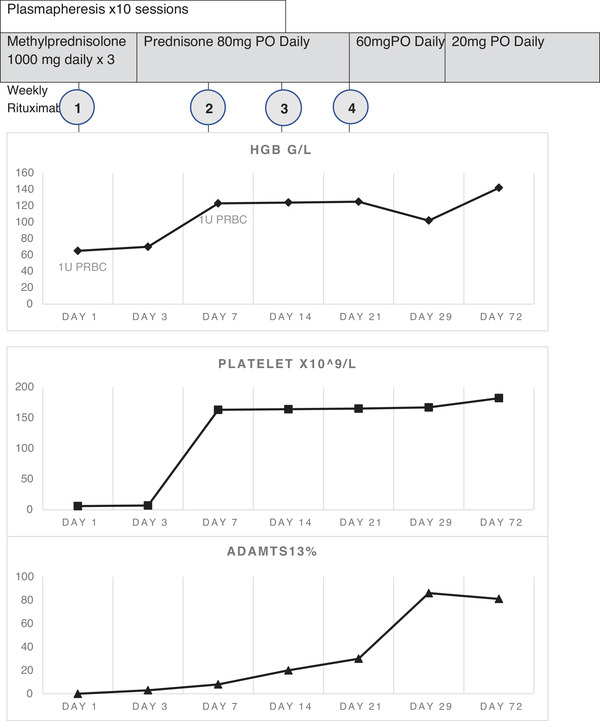
Laboratory improvement following corticosteroids, plasmapheresis and rituximab therapy

He received corticosteroids, rituximab, and a total of 12 plasma exchange sessions over the course of 2 weeks until platelet counts remained above 150 × 10^9^/L. He continued the treatment of a total of four doses of weekly rituximab and prednisone 80 mg daily. He returned to South Korea where he works on day 29 after the diagnosis of TTP. On Day 142, he reported from South Korea that he was doing well, on prednisone 20 mg daily and ADAMTS13 was 97.1%. (Figure 2).

## 3 DISCUSSION

We present a case of a 61‐year‐old male patient with no past medical history who developed TTP 5 days after the first dose of Pfizer COVID‐19 vaccination. He was treated with plasmapheresis, corticosteroids and rituximab with a rapid and excellent response. Given the clinical presentation, lab results and lack of thrombosis, Vaccine‐induced TTP was less likely and TTP linked to COVID‐19 vaccination was the most likely diagnosis.

To the best of our knowledge, this is the second report of a de novo TTP following mRNA Pfizer COVID‐19 vaccination, and the third report of aTTP following COVID‐19 vaccination. De Bruijn et al. [2] reported the first case of a 38‐year‐old woman with TTP at 2 weeks following the first dose of Pfizer COVID vaccine. Al‐Ahmad et al. [3] reported a case of a 37‐year‐old man who developed TTP at 10–15 days after the AstraZeneca‐Oxford COVID‐19 vaccination. Also, a few other cases of TTP development following Pfizer and AstraZeneca‐Oxford COVID‐19 vaccination are mentioned in Table 1.

**TABLE 1:** Description of a few cases of TTP after COVID‐19 vaccination that have been reported

**Reference**	**Types of vaccine**	**Age of patient and days of TTP symptom onset following vaccine dose(s)**	**ADAMTS13 level at diagnosis**	**Treatment**	**Outcome/survival**
[2]	BNT162b2 mRNA COVID‐19 vaccine (Pfizer)	38 yo F; 3 weeks after the second dose	0	17 sessions of plasma exchange, corticosteroids, rituximab,, caplacizumab	Discharged with ADAMTS13 activity 0%
[6]	BNT162b2 mRNA COVID‐19 vaccine (Pfizer)	69 yo M; 7 days after the first dose	<2%	5 sessions of plasma exchange prednisone, 1 dose of rituximab	Discharged and received four additional doses of Rituximab
[3]	AstraZeneca‐Oxford COVID‐19 vaccine	37 yo M; 10–15 days after the first dose	2.6%	8 sessions of plasma exchange,methylprednisolone 1 g x 3 days followed by prednisolone 1 mg/kg	Discharged and received 4 doses of rituximab. Complete remission
[7]	BNT162b2 mRNA COVID‐19 vaccine (Pfizer)	4o yo F 8 days after the second dose 28 yo M 28 days after the second dose 31 yo F 13 days after the first dose (relapsed TTP) 30 yo M 8 days after the second dose (relapsed TTP)	0 0 0 0	6 sessions of plasma exchange, high dose steroids, caplacizumab 5 sessions of plasma exchange, caplacizumab, high dose steroids, rituximab 4 sessions of plasma exchange, steroids, rituximab, caplacizumab 5 sessions of plasma exchange, steroids, rituximab, caplacizumab	Complete remission Complete remission 10 weeks after treatment, ADAMTS13 activity is 0%, continues caplacizumab Complete remission

Vaccination in general is an important part of preventive medicine. Rarely vaccination, especially against viral infection, such as influenza vaccine [4], has been associated with autoimmune pathology including acquired TTP.

The mechanism linking TTP with COVID‐19 vaccines is poorly understood. Similar to natural infections, flu vaccines may induce anti‐ADAMTS13 antibodies. Sobolev et al. showed that within 24 h of receiving an adjuvanted swine flu vaccine, healthy individuals made expansive and complex molecular and cellular responses [5]. It is possible that the wide‐ranging adverse clinical events associated with vaccination are immunological correlates. The association of the rise of anti‐ADAMTS13 autoantibodies by COVID‐19 vaccines warrants additional research.

This case report suggests the potential, but not a proven role, of mRNA Pfizer COVID‐19 vaccination in the pathogenesis of aTTP. Vaccine‐related aTTP, like other forms of aTTP, can be successfully treated by prompt initiation of plasmapheresis, corticosteroids and/or rituximab. Further studies are needed to verify the possible association between microangiopathic thrombotic disorders following the administration of vaccines against COVID‐19.

## CONFLICT OF INTEREST

The authors declare that they have no conflict of interest.
